# Early percutaneous antegrade—retrograde rendezvous repair of a transected ureter—a real therapeutic option

**DOI:** 10.1093/jscr/rjac465

**Published:** 2022-11-08

**Authors:** Christopher Garvey, Pat Rohan, Ronan Motyer, Michael Courtney, J Mark Ryan

**Affiliations:** Department of Interventional Radiology, St. James’s Hospital, Dublin, Ireland; Department of Interventional Radiology, Beaumont Hospital, Dublin, Ireland; Department of Interventional Radiology, St. James’s Hospital, Dublin, Ireland; Department of Interventional Radiology, St. James’s Hospital, Dublin, Ireland; Department of Interventional Radiology, St. James’s Hospital, Dublin, Ireland

## Abstract

A 46-year-old female underwent elective laparoscopic hysterectomy. Seven days post-operatively, she presented with urinary leak from the vagina. Computed tomography urogram demonstrated a right complete ureteric transection with leakage of urine into the pelvis and fistulation into the vagina. A rendezvous procedure was performed via a retrograde cystoscopic approach during which a guidewire was used to cannulate the right ureteric orifice and coiled in the retroperitoneal cavity. Subsequently, via a right percutaneous nephrostomy, a guidewire was advanced through the site of ureteric transection, which was followed by a snare catheter to bring the retrograde wire externally. A nephroureteric stent was then inserted. Twelve weeks later, the nephroureteric stent was exchanged for a ureteric stent for 6 months. A subsequent retrograde ureterogram showed complete healing of the ureter. The ureteric stent was removed and follow-up ultrasounds revealed no hydronephrosis. Percutaneous rendezvous procedures represent an effective option to treat this challenging condition.

## INTRODUCTION

Ureteric injury during hysterectomy represents a significant cause of morbidity [1]. The incidence of urinary tract injury during gynaecological surgery is estimated to be between 0.3 and 1% [2, 3]. Of all urinary tract injuries, 75% are iatrogenic relating to gynaecological surgery [3].

The majority of ureteric injuries caused during hysterectomy are diagnosed post-operatively [5]. While all urinary tract injuries increase morbidity, post-operative diagnosis results in significant increase in potentially life-threatening complications such as sepsis and acute renal failure [4].

We present the case of a 46-year-old female who experienced a complete ureteric transection during laparoscopic abdominal hysterectomy and bilateral salpingo-oophrectomy which were diagnosed 2 weeks post-operatively. The patient underwent a hybrid rendezvous procedure to repair the transected ureter.

## CASE REPORT

A 46-year-old female was admitted electively under the Gynaecology service for elective laparoscopic abdominal hysterectomy and bilateral salpingo-oophrectomy. She had a history of refractory endometriosis. Her past surgical history included multiple laparoscopies for the investigation and treatment of same.

No injury was identified intraoperatively. She had an uneventful immediate post-operative course and was discharged home on the fourth post-operative day. On post-operative day 7, she presented with a 6-day history of continuous urinary incontinence.

**Figure 1:**
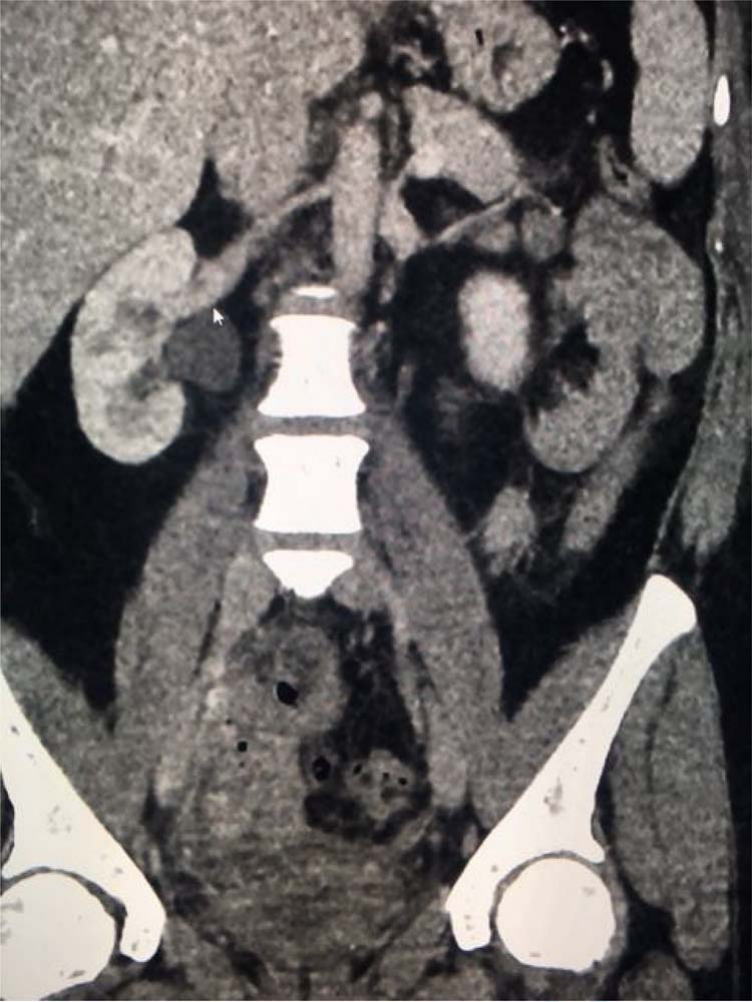
Coronal nephrogenic phase CT urogram showing hydronephrosis of the right renal pelvis.

**Figure 2:**
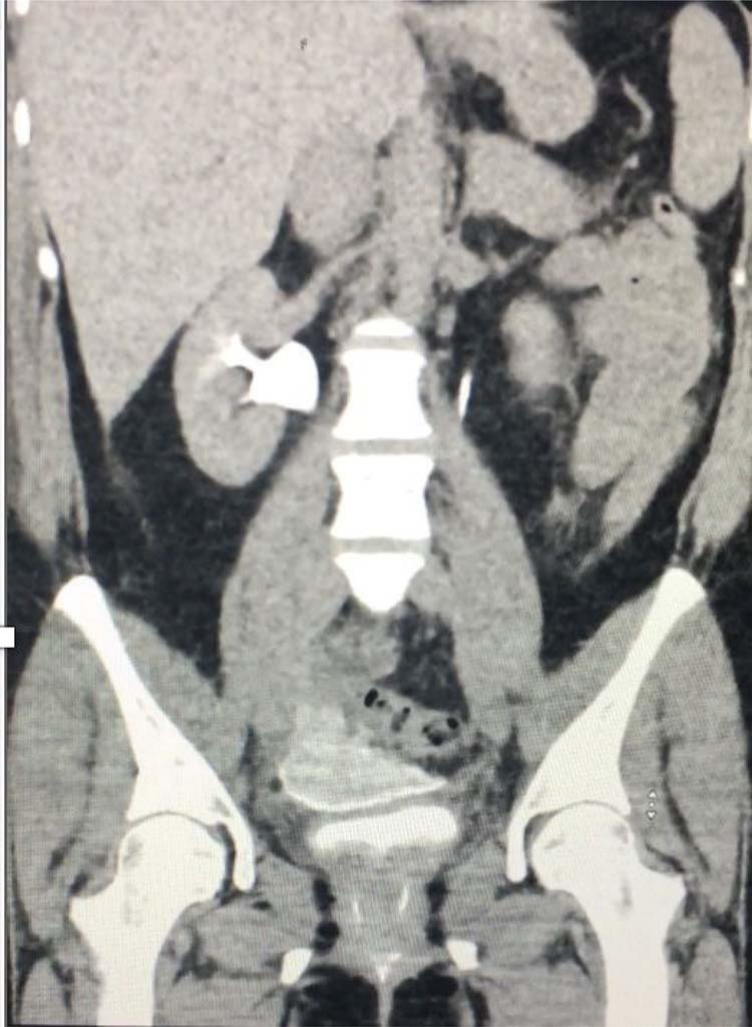
Coronal delayed phase CT urogram showing pooling of contrast in the pelvis.

**Figure 3:**
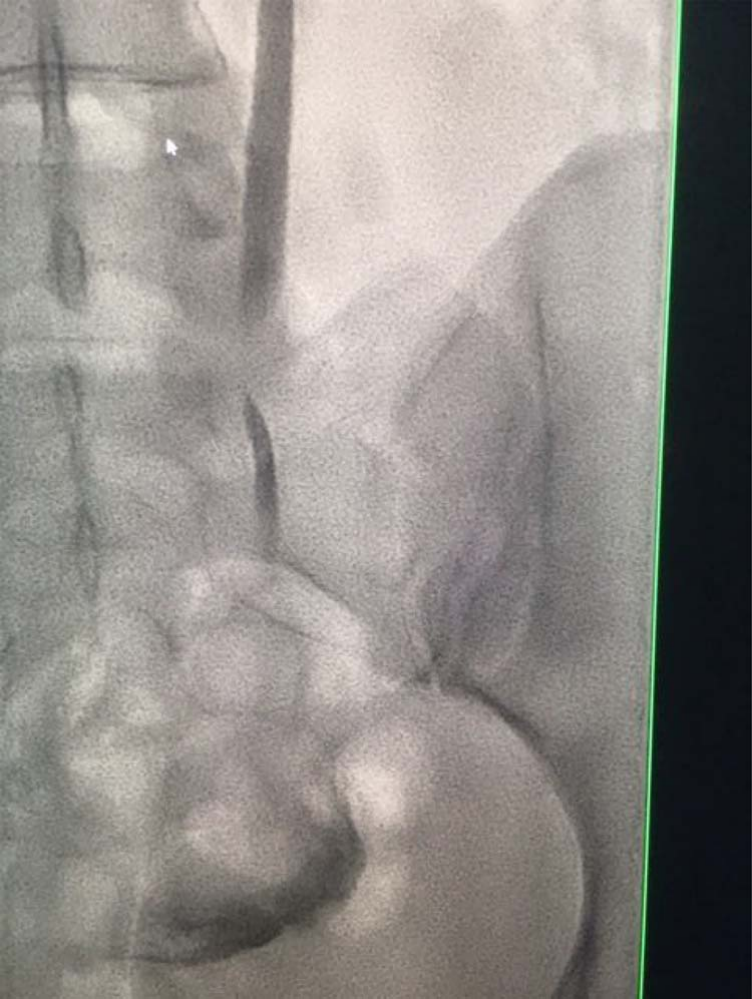
Antegrade urethrogram showing proximal ureter opacification with contrast, with an abrupt end point consistent with transected right distal ureter.

Computed tomography (CT) with urographic phase imaging demonstrated a right distal ureteric injury with leakage of urine into the pelvis and fistulation into the vagina (Figs 1 and 2). A diverting right percutaneous nephrostomy was inserted in the interventional radiology (IR) suite. An antegrade ureterogram at the same time demonstrated a complete discontinuation of the ureter which appeared retracted (Fig. 3).

Following a discussion between the interventional radiologist and the urologist, the patient underwent a combined endoscopic and radiologic rendezvous procedure 2 days later. Via a retrograde cystoscopic approach, a 250-cm, 0.018-inch guidewire was used to cannulate the right ureteric orifice and was passed through the distal right ureter into the retroperitoneal cavity where it was coiled. The guidewire access was secured and the patient was then transferred to the IR suite, placed in the prone position.

**Figure 4:**
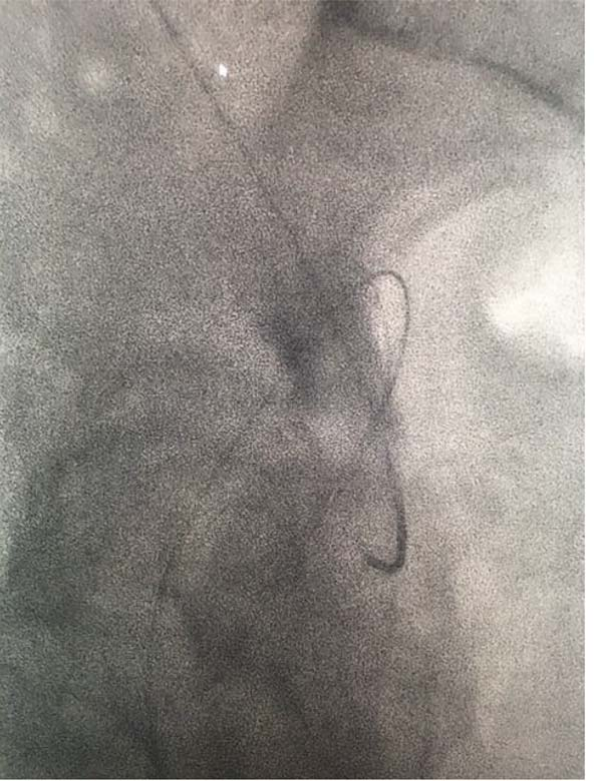
Fluoroscopic images showing the antegrade Amplatz gooseneck snare catheter attempting to grasp the retrograde wire.

**Figure 5:**
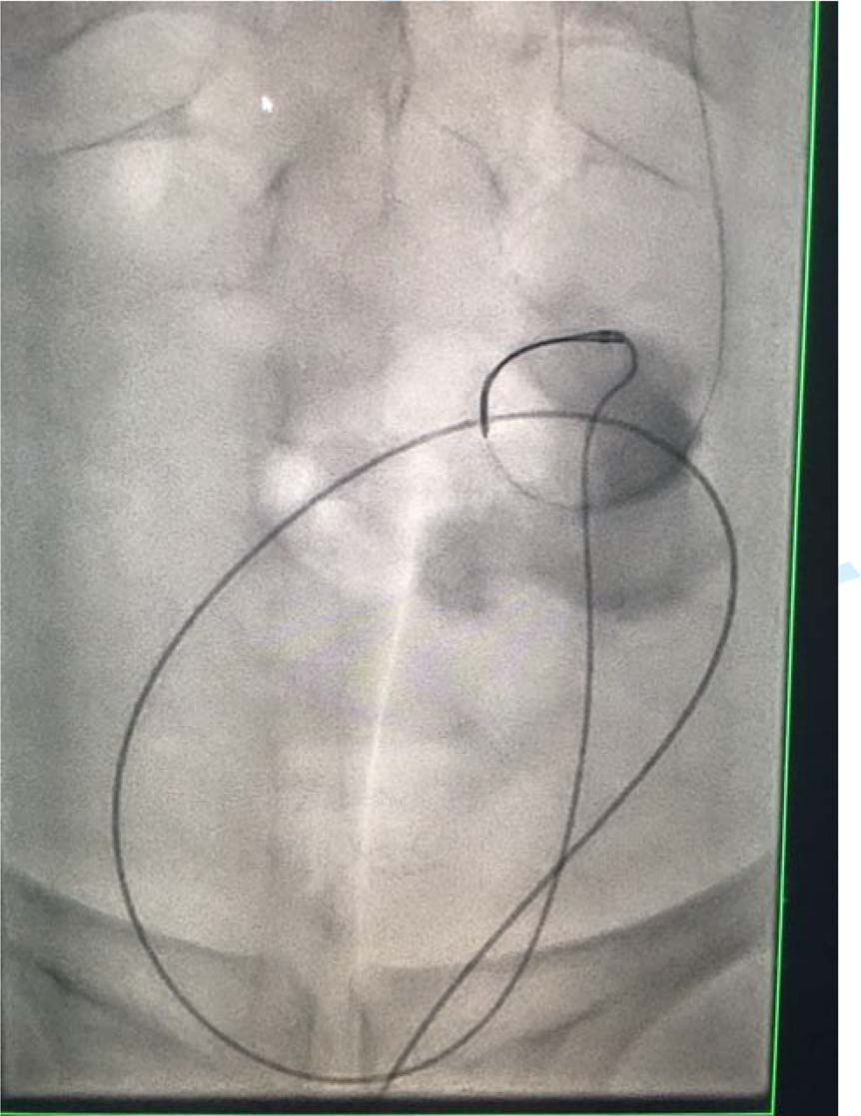
Fluoroscopic images showing the antegrade Amplatz gooseneck snare catheter attempting to grasp the retrograde wire.

**Figure 6:**
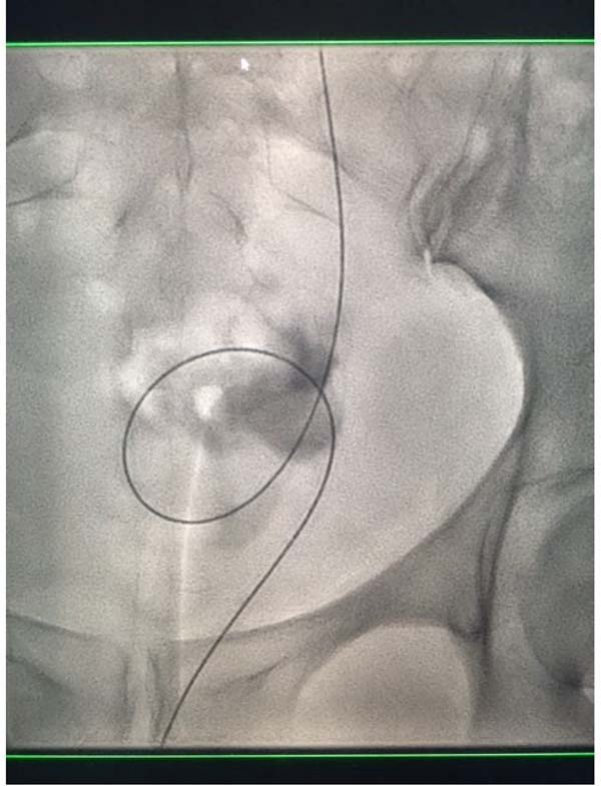
Fluoroscopic images showing the retrograde wire successfully retrieved and brought externally via the nephrostomy site, achieving through-and-through wire access.

**Figure 7:**
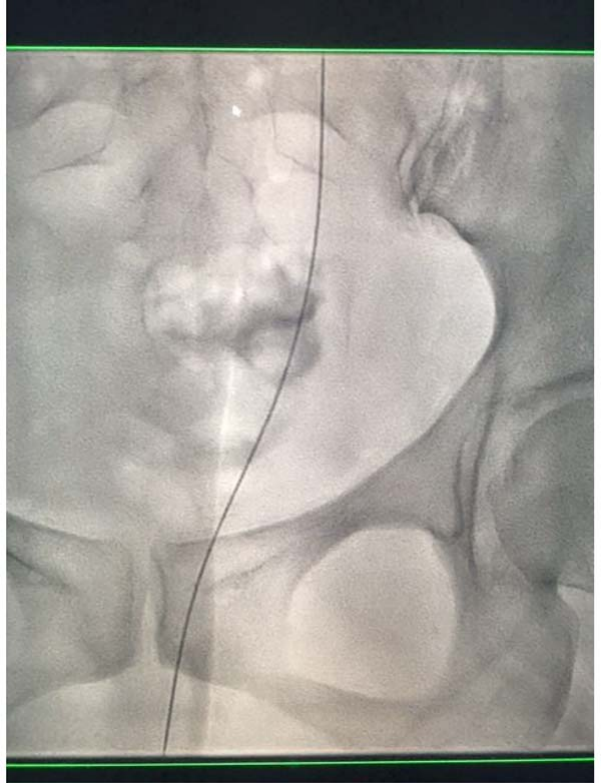
Fluoroscopic images showing the retrograde wire successfully retrieved and brought externally via the nephrostomy site, achieving through-and-through wire access.

Under conscious sedation, using the previously inserted right percutaneous nephrostomy, antegrade access was achieved into the intrarenal collecting system and then into the proximal ureter. An 8 Fr × 11-cm access sheath was introduced. A guidewire was advanced antegradely through the site of ureteric transection and coiled in a position adjacent to the position of the guidewire that had been inserted retrogradely. A Gooseneck snare catheter was then passed over the guidewire and the snare was inserted (Figs 4 and 5). Under fluoroscopic guidance, the retrograde wire was retrieved and brought externally via the nephrostomy site, achieving through-and-through wire access (Figs 6 and 7).

**Figure 8:**
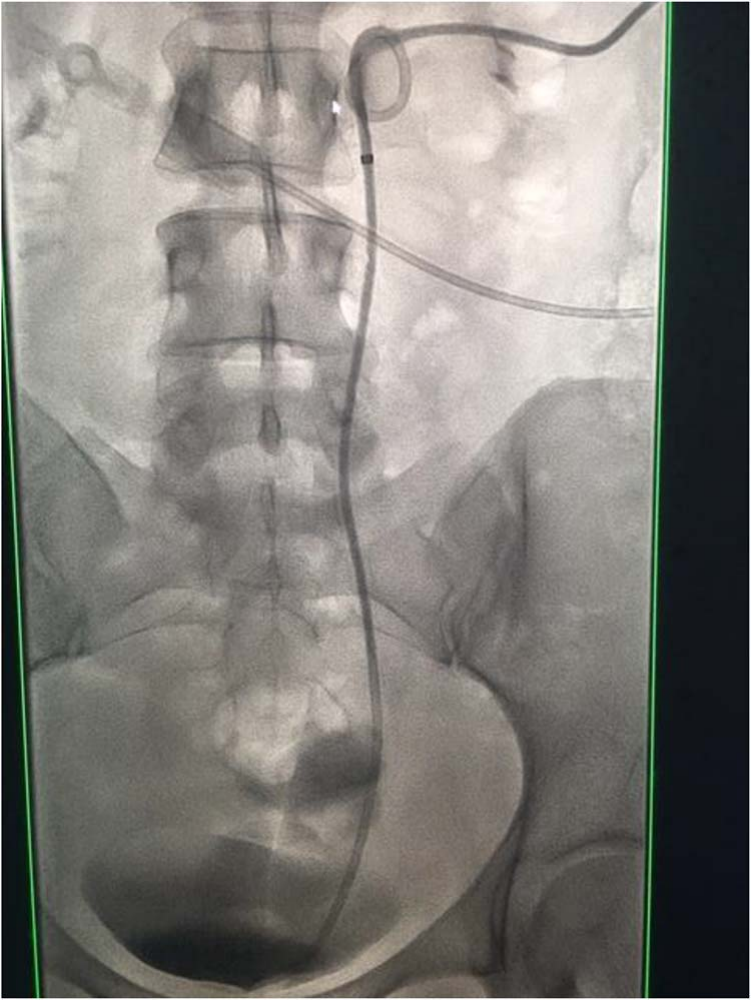
Fluoroscopic images demonstrating the successfully re-connected right ureter with a nephroureteric stent *in situ*.

A catheter-guidewire exchange was performed to upsize the guidewire to a 0.035-inch stiff guidewire. An 8-Fr, 26-cm nephroureteric stent was inserted over the guidewire in an antegrade manner (Fig. 8). The stent was left to free external drainage. The patient was transferred back to the ward and discharged home the following day. Trial without bladder catheter was successfully undertaken 1 week post-procedure.

**Figure 9:**
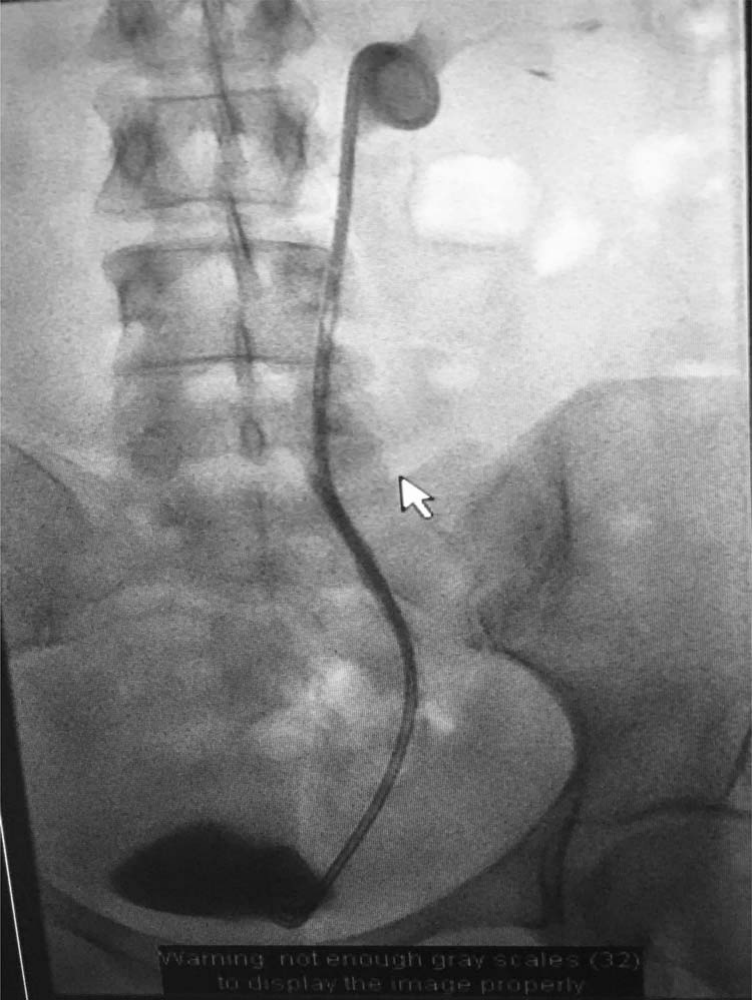
Fluoroscopic image showing ureteric stent.

An antegrade ureterogram performed 6 weeks post-procedure demonstrated partial healing of the ureter but persistent contrast extravasation was present, so the nephroureteric stent was replaced. Six weeks later, an antegrade ureterogram demonstrated excellent healing and the leak had resolved, so the nephroureteric stent was exchanged for a 24-cm, 8-Fr antegrade ureteric stent (Fig. 9).

**Figure 10:**
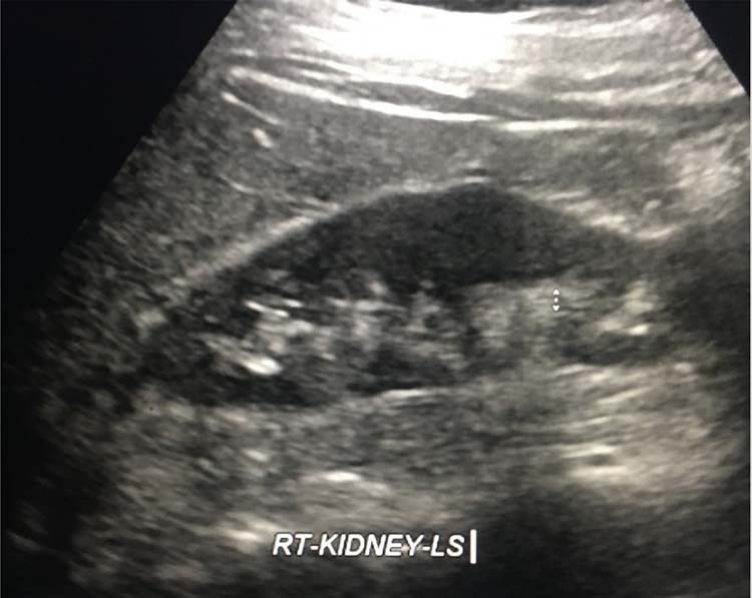
Follow-up US kidneys revealed no hydronephrosis.

Three months later, the Urology department performed a retrograde ureteric stent exchange. Five months later, IR removed the ureteric stent via a retrograde approach. Retrograde ureterogram showed a fully healed, intact ureter. The ureteric stent was therefore not replaced. Three months later, follow-up ultrasound (US) showed no evidence of the obstruction (Fig. 10).

## DISCUSSION

Ureteric injuries are uncommon, but most are iatrogenic occurring during gynaecological, colorectal and vascular surgeries [5, 6]. Gynaecological surgery accounts for 52–82% of all iatrogenic ureteric injuries [7]. Iatrogenic injuries are detected intraoperatively in 8.6% of cases, with most diagnosed post-operatively when patients develop symptoms of complications [5]. The gold standard for diagnosis is CT urography, which may demonstrate contrast extravasation in addition to information for therapeutic planning. These injuries present significant therapeutic challenges and controversy exists regarding the best approach [7–10].

Ureteric injuries may present a complex therapeutic challenge due to dense structuring or a tortuous ureter. This often necessitates multiple attempted procedures [8]. Surgical repair by open, laparoscopic and robotic-assisted approaches have been described [10]. Percutaneous approaches were first described in 1984 by Druy *et al*. [11], and it has been demonstrated that minimally invasive treatment options, including percutaneous nephrostomy, ureteric stenting or both, can be effective in up to 80% [11, 12].

Minimally invasive techniques utilizing both antegrade and retrograde access, so-called rendezvous procedures, have been shown to improve success in technically difficult cases [8, 13]. This technique was first described by Watson *et al*. in 2002 [14]. They were successful in crossing the injured ureter in all 20 cases. In small series published to date, rendezvous approaches have had success in restoring ureteric continuity with low morbidity rates [7]. Pastore *et al*. [15] demonstrated 66% success rate in restoring ureteric continuity without leakage. Ureteric stricture was noted in 22% and persistent leakage was noted in 12% [10].

Two types of rendezvous procedures have been described. These include endoscopic rendezvous, combined antegrade flexible ureterorendoscopic and retrograde semi-rigid ureteroscopic approaches [10, 16]. Long-term success rates of up to 78% have been reported for endoscopic rendezvous repairs of transected ureters [10].

The second technique involves combined endoscopic and radiologic approaches. Retrograde cystoscopic insertion of a guidewire and antegrade access via fluoroscopy in an IR suite [8, 9]. This technique has more commonly been used in cases of complex ureteric stricture rather than transection [9].

Inter-departmental collaboration may reduce the number of separate procedures for the patient and result in a more timely successful outcome. In our case, the combined radiologic and endoscopic rendezvous approach yielded a successful outcome. The interventional radiologist plays a crucial role in diagnosis and treating these complex iatrogenic injuries in a safe and effective manner. Further prospective studies are required to support the early promising data.

## CONFLICT OF INTEREST STATEMENT

The authors have no conflict of interest to declare.
